# Efficacy and safety of probiotic/synbiotic supplementation for osteoporosis: a meta-analysis of randomized controlled trials

**DOI:** 10.3389/fmed.2026.1731528

**Published:** 2026-02-03

**Authors:** Xinyu Wang, Lei Zhou, Xingming Yu, Qiang Hou, Chenglong Wang, Wei Cui, Yuheng Hu, Xiumei Wang, Zhuangchen Zhu

**Affiliations:** 1 Department of Orthopaedic Surgery, The Second Affiliated Hospital of Shandong First Medical University, Tai’an, Shandong, China; 2 Department of Burn and Plastic Surgery, The Second Affiliated Hospital of Shandong First Medical University, Tai’an, Shandong, China; 3 Department of Pediatric Surgery, The Second Affiliated Hospital of Shandong First Medical University, Tai’an, Shandong, China

**Keywords:** meta-analysis, osteoporosis, probiotics, RCTs, synbiotics

## Abstract

**Objectives:**

To evaluate the efficacy and safety of probiotic/synbiotic supplementation for osteoporosis.

**Methods:**

PubMed, Embase, Web of Science, and Cochrane databases were used to screen studies up to October 2025. Data pooling used standardized mean differences (SMD) or risk ratios (RR) with 95% confidence intervals (CI). Sensitivity analysis assessed result stability. Review Manager 5.4 and STATA 15.1 were used to analyze. Publication bias was assessed by Egger’s test and funnel plots. Evidence for each outcome was evaluated and graded according to GRADE.

**Results:**

Ten randomized controlled trials (RCTs) with 732 patients were included. Significant improvements in lumbar spine bone mineral density (BMD) (SMD: 0.85; 95% CI: 0.01, 1.69; *P* = 0.049) and parathyroid hormone (SMD: −1.21; 95% CI: −2.19, −0.23; *P* = 0.02) existed in the probiotic/synbiotic group. No increase in adverse event risk was observed (RR: 1.03; 95% CI: 0.86, 1.23; *P* = 0.78). No significant effects were found on total hip BMD, osteocalcin, C-terminal telopeptide, alkaline phosphatase, or osteoprotegerin. No publication bias was detected.

**Conclusion:**

Probiotic/synbiotic supplementation may be safe and effective as an adjunctive treatment for osteoporosis, improving bone density without increasing adverse reactions. Larger, multicenter RCTs are needed to confirm these findings.

**Systematic Review Registration:**

https://www.crd.york.ac.uk/PROSPERO/view/CRD42024540614, identifier CRD42024540614.

## 1 Introduction

Osteoporosis is a systemic bone disease characterized by low bone mass, damaged bone microstructure, increased bone fragility, and a high risk of fractures (1, 2). Its incidence continues to rise with the aging population, becoming a serious public health challenge worldwide. According to the latest data from the International Osteoporosis Foundation, approximately 200 million people worldwide suffer from this disease, resulting in more than 8.9 million fractures annually–an osteoporotic fracture every 3 s on average (3, 4). Therefore, prevention and treatment strategies for osteoporosis must be proactive, focusing on early diagnosis and intervention to effectively prevent the first fracture. However, existing major treatments, including basic measures such as calcium and vitamin D supplementation, as well as anti-resorption drugs (such as bisphosphonates and RANKL inhibitors like denosumab) and bone-forming drugs (such as parathyroid hormone analogs like teriparatide), while reducing fracture risk to some extent, still face many limitations in their clinical application (5, 6). For example, long-term use of bisphosphonates may increase the risk of atypical femoral fractures and osteonecrosis of the mandible (7); while estrogen receptor modulators increase the risk of venous thromboembolic events (8). Furthermore, these drugs are often expensive and require long-term or even lifelong administration, posing significant challenges to patient adherence management (9). Many patients frequently discontinue treatment due to side effects, cost, or inconvenience of administration, resulting in a substantial reduction in efficacy (10). These treatment challenges highlight the urgent need to explore novel, safe, economical, and easily sustainable adjuvant osteoporosis treatment strategies. In recent years, gut-bone axis-targeting microecological interventions–especially the use of probiotics and synbiotics–have injected new vitality into this field (11).

Probiotics are defined as “live microorganisms that, when ingested in adequate amounts, produce beneficial effects on the health of the host,” (12) while synbiotics refer to a mixture of probiotics and prebiotics that probiotics can selectively utilize to promote the health of the host (13). Animal studies have provided strong support for the bone-protective effects of probiotics; for example, supplementing ovariectomized mice with strains of *Lactobacillus* and *Bifidobacterium* significantly alleviates bone loss (14). However, when the research focus shifts to human clinical trials, the evidence presents considerable heterogeneity and contradiction. Several newly published randomized controlled trials (RCTs) have reached inconsistent conclusions, with some studies showing no significant effect of probiotics/synbiotics on hip bone mineral density or specific bone turnover markers (15–17). These controversies leave the exact efficacy and safety of probiotics/synbiotics in osteoporosis management unresolved, hindering their translation into clinical practice. The inconsistencies in conclusions may stem from significant differences among studies in strain types, dosages, intervention durations, and baseline characteristics of study subjects, all of which constitute significant sources of clinical heterogeneity.

Therefore, this study aims to address the uncertainties in the current evidence framework through an updated and more comprehensive systematic review and meta-analysis. Compared to the work of Zeng et al. (18), the innovation and value of this study are reflected in the following aspects: first, the timeliness is updated. We have expanded the scope of the literature search to October 2025, maximizing the inclusion of the latest published clinical randomized controlled trial evidence to ensure that the conclusions reflect the latest research progress. Second, the scope is expanded. For the first time, we have included synbiotic intervention in the analysis and systematically evaluated the impact of probiotics/synbiotics on a wider range of outcome indicators, including lumbar and hip bone mineral density (BMD), a series of key bone turnover markers [osteocalcin, C-terminal telopeptide (CTX), alkaline phosphatase, osteoprotegerin, parathyroid hormone], and the incidence of adverse events, thereby providing a more comprehensive efficacy and safety profile. In summary, this study aims to clarify the key clinical question of whether probiotic/synbiotic supplements can serve as a safe and effective adjunctive therapy to improve bone mineral density, regulate bone metabolism, and avoid additional risks in patients with osteoporosis by integrating the latest high-quality evidence up to October 2025. The goal is to provide the highest level of evidence-based medicine for future clinical practice guidelines and research directions.

## 2 Methods

### 2.1 Literature search

This study was carried out following the PRISMA 2020 guidelines (19). The registration ID is CRD42024540614 in PROSPERO. We systematically searched PubMed, Embase, Web of Science, and Cochrane up to October 2025 evaluating the efficacy and safety of probiotic/synbiotic supplementation for osteoporosis. Search terms included “Probiotics,” “Synbiotics,” and “Osteoporosis.” The detailed PubMed search strategy is as follows: (((“Probiotics”[Mesh]) OR (Probiotic)) OR ((“Synbiotics”[Mesh]) OR (Synbiotic))) AND ((“Osteoporosis”[Mesh]) OR ((((((((((Osteoporoses) OR (Post-Traumatic Osteoporoses)) OR (Post-Traumatic Osteoporosis)) OR (Senile Osteoporoses)) OR (Senile Osteoporosis)) OR (Age-Related Bone Loss)) OR (Age-Related Bone Losses)) OR (Age-Related Osteoporosis)) OR (Age Related Osteoporosis)) OR (Age-Related Osteoporoses))). We also screened the bibliographies of RCTs. Two researchers screened the titles and abstracts for retrieving eligible articles independently, and resolved discrepancies through discussion. The search strategy was depicted in Supplementary Table 1.

### 2.2 Inclusion and exclusion criteria

Participants in this study were patients with osteoporosis. The intervention was probiotic/synbiotic supplementation, while the control group consisted of placebo or standard treatment. Outcome measures of interest included lumbar spine and total hip bone mineral density, bone turnover markers, and adverse events. The study type was limited to randomized controlled trials. Exclusion criteria included: study protocols, unpublished studies, non-original studies, non-RCT studies, studies with insufficient data, and review articles.

### 2.3 Data abstraction

Two authors conducted the data extraction independently, with discrepancies resolved by another investigator. First author, publication year, study region, design, registration number, intervention, control, sample size, age, BMI, intervention duration, lumbar spine BMD, total hip BMD, alkaline phosphatase, osteocalcin, CTX, osteoprotegerin, parathyroid hormone, and adverse events were all extracted. Complete dataset would be retrieved from corresponding authors for addressing the insufficient problem.

### 2.4 Quality evaluation

Randomized controlled trials’ quality was assessed using the Cochrane Handbook for Systematic Reviews of Interventions 5.1.0 via seven domains: sequence generation randomization, allocation concealment, blinding of participants and personnel, outcome assessment blinding, incomplete outcome data, selective outcome reporting, and other potential biases (20). Each domain was rated as low risk, high risk, or unclear risk. Studies with more “low risk” ratings were considered of higher quality. Two authors evaluated the studies’ quality, resolving disagreements through discussion.

### 2.5 Statistical analysis

Review Manager 5.4.1 was used to analyze. Standardized mean differences (SMD) with 95% confidence intervals (CI) were used for continuous outcomes. Risk ratios (RR) with 95% CI were applied for dichotomous data. Heterogeneity was evaluated with the chi-squared (χ^2^) test (Cochran’s Q) and the inconsistency index (21). Substantial heterogeneity was defined by a χ^2^ *P*-value < 0.1 or an I^2^ > 50%. The overall SMD or RR was calculated using a random-effects model. For results with more than two studies, a sensitivity analysis was conducted. Funnel plots and Egger’s regression tests were used for evaluating publication bias (22) in Stata 15.1 (Stata Corp., College Station, Texas, USA), with a *P*-value < 0.05 indicating statistically. When the number of included studies is ≥10, Egger’s regression test is used to assess publication bias; if the number of included studies is <10, visual inspection is performed only using a funnel plot. Additionally, according to GRADE, each outcome’s evidence was evaluated and graded as “high,” “moderate,” “low,” or “very low” quality (23).

## 3 Results

### 3.1 Literature retrieval, study characteristics, and baseline

**FIGURE 1:**
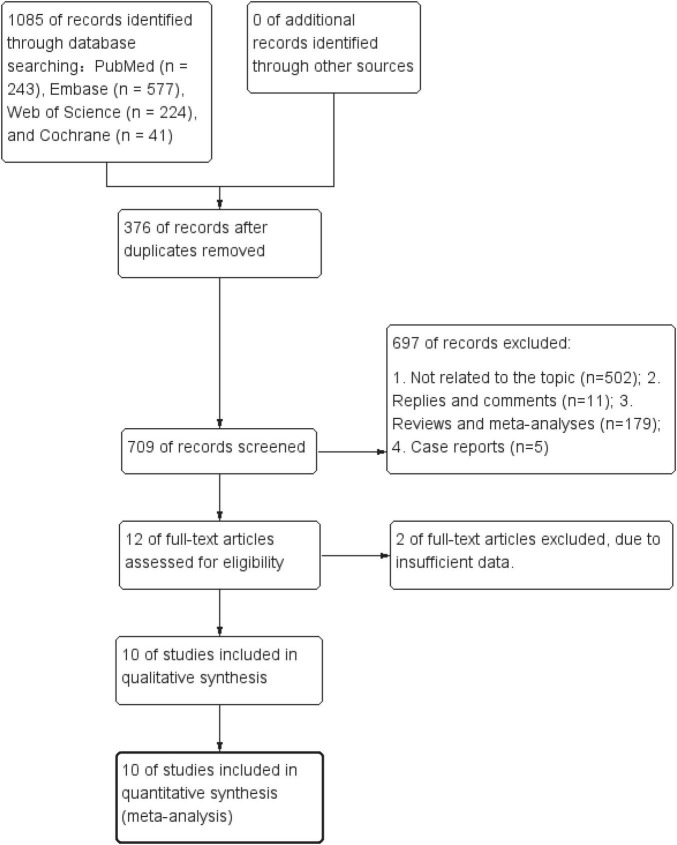
Flowchart of the systematic search and selection process.

**FIGURE 2:**
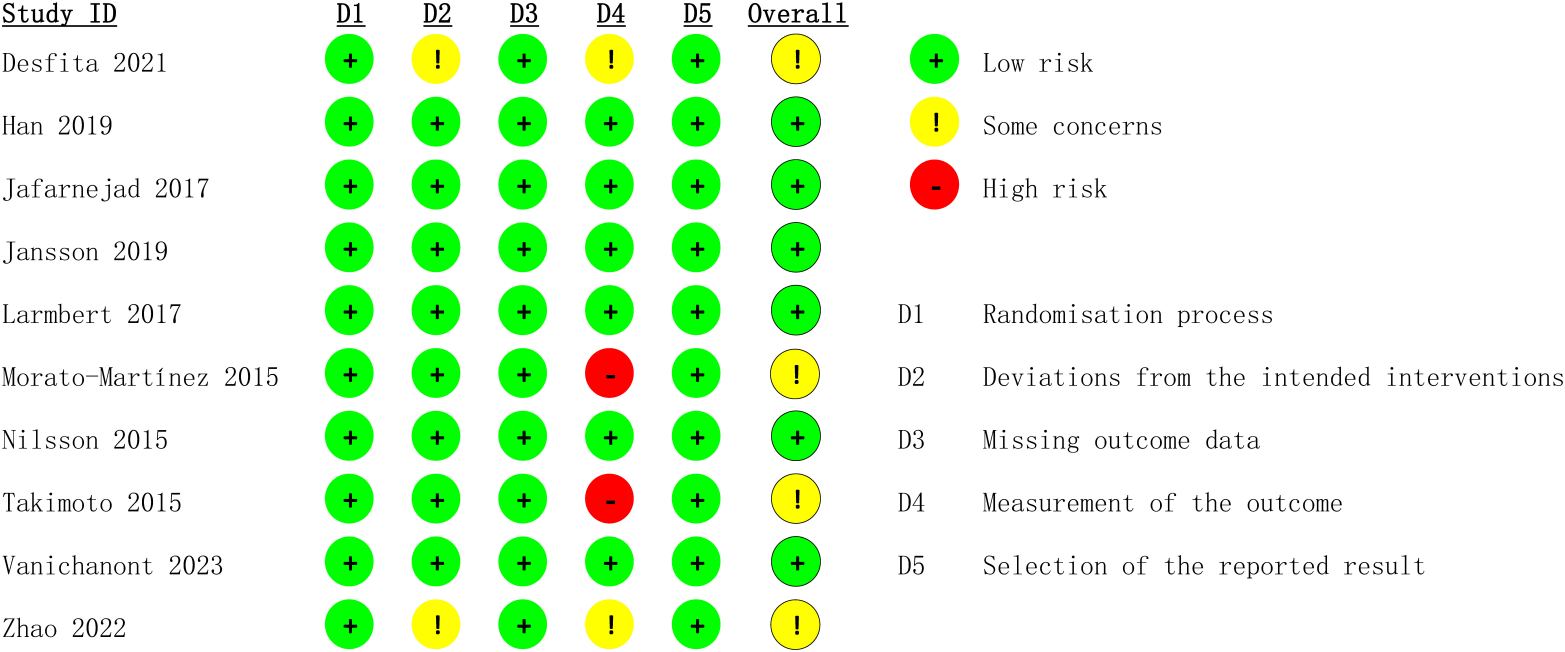
Details of the quality evaluation for included RCTs.

Figure 1 showed the literature screening process. A total of 1,085 articles from PubMed (*n* = 243), Embase (*n* = 577), Web of Science (*n* = 224), and Cochrane (*n* = 41) were identified. After removing duplicates, 709 titles and abstracts were reviewed, and 10 RCTs (15–17, 24–30) involving 732 patients were included. Table 1 lists the characteristics of studies, and Figure 2 presents the quality evaluation results. These studies, published between 2013 and 2023, were conducted in multiple locations worldwide, including Sweden (2 studies), China (1 study), Thailand (1 study), Iran (1 study), South Korea (1 study), Indonesia (1 study), Japan (1 study), Spain (1 study), and Denmark (1 study). All studies employed a randomized controlled design, with 7 studies prospectively registered on international clinical trial registries such as ClinicalTrials.gov.

**TABLE 1:** Characteristics of included studies.

References	Year	Country	Study design	Registration number	Population	Intervention	Control
Jansson et al. (17)	2019	Sweden	RCT	NCT02722980	Postmenopausal women with osteoporosis	*Lactobacillus paracasei*, *Lactobacillus plantarum* and *Lactobacillus plantarum*	Placebo
Nilsson et al. (25)	2015	Sweden	RCT	NCT02422082	Women from the population who were 75 to 80 years old and had low BMD	*Lactobacillus reuteri*	Placebo
Zhao et al. (29)	2022	China	RCT	ChiCTR1800019268	Postmenopausal women with osteoporosis	*Lactobacillus*, *Bifidobacterium*	Placebo material, calcium, calcitriol
Vanichanont et al. (15)	2023	Thailand	RCT	TCTR20230326002	Postmenopausal women with osteoporosis	*Lactobacillus reuteri*, *Lactobacillus paracasei*, *Lactobacillus rhamnosus*, *Lactobacillus rhamnosus*, *Lactobacillus animalis*, *Lactobacillus longum lactobacillus. longum OLP-01*	270 mg of inulin
Jafarnejad et al. (26)	2017	Iran	RCT	IRCT2015092024103N1	Postmenopausal women with osteoporosis	*Lactobacillus casei*, *Bifidobacterium longum*, *Lactobacillus acidophilus*, *Lactobacillus rhamnosus*, *Lactobacillus bulgaricus*, *Bifidobacterium brevis*, *Streptococcus thermophilus*	500 mg of corn starch
Han et al. (16)	2019	Korea	RCT	IRB B1904-532-004	Postmenopausal women with osteoporosis	*Lactobacillus*	placebo
Desfita et al. (30)	2021	Indonesia	RCT	98/EA/KEPK/2020	Postmenopausal women with osteoporosis	*Lactobacillus casei*	Soybean milk
Takimoto et al. (24)	2015	Japan	RCT	NA	Postmenopausal women with osteoporosis	*Bacillus subtilis*	Placebo
Morato-Martínez et al. (26)	2015	Spain	RCT	NCT02629341	Menopausal women with osteoporosis	Dairy products rich in bioactive nutrients (*L-leucine* and the probiotic *Lactobacillus plantarum*)	Eat one serving of the same product, but not fortified
Lambert et al. (27)	2013	Denmark	RCT	NCT02174666	Postmenopausal women with osteoporosis	Isoflavones combined with probiotics	90 l of water and 250 g of brown food coloring
**Treatment time**	**Sample size**		**Age (years)**		**BMI (kg/m^2^**)		**Outcomes**
	**Intervention**	**Control**	**Intervention**	**Control**	**Intervention**	**Control**	
6 months	116	118	59.1 ± 3.8	58.1 ± 4.3	24.2 ± 2.7	23.9 ± 2.6	F1; F8
12 months	34	36	76.4 ± 1.0	76.3 ± 1.1	25.5 ± 3.5	25.3 ± 3.3	F1; F2; F8
3 months	15	12	62.77 ± 6.00	61.63 ± 7.86	23.13 ± 2.19	23.37 ± 2.53	F3; F4; F7
6 months	20	20	62 ± 5.07	64.05 ± 3.58	23.35 ± 3.77	24.20 ± 2.78	F5; F7
6 months	20	21	58.85 ± 0.68	57.29 ± 0.72	24.86 ± 0.41	23.82 ± 0.38	F2; F4; F5; F6; F7
6 months	27	26	58.4 ± 3.4	59.5 ± 3.4	24.3 ± 2.6	23.0 ± 2.0	F4; F5
3 months	35	20	55.88 ± 4.53	65.16 ± 8.25	27.44 ± 4.19	25.51 ± 6.19	F4
6 months	34	35	57.5 ± 4.3	57.8 ± 5.4	22.2 ± 3.3	22.1 ± 2.7	F1; F2
6 months	33	32	NA		NA		F3; F5; F7
12 months	38	40	60.84 ± 1.07	62.85 ± 0.99	24.84 ± 0.62	26.65 ± 0.81	F4; F5; F6

F1, change of lumbar spine; F2, change of total hip; F3, alkaline phosphatase; F4, Osteocalcin (OC); F5, C-terminal telopeptide (CTX); F6, OPG; F7, parathyroid hormone; F8, any adverse event.

The patient populations included in the studies were highly homogeneous, all with a confirmed diagnosis of osteoporosis. Nine studies specifically targeted postmenopausal women. Baseline patient characteristics showed that the mean age of the overall sample ranged from 50 to 76 years, with most studies showing a mean age of 57–62 years, reflecting the high prevalence of osteoporosis in middle-aged and older women. One study from Sweden [Nilsson et al. (25)] focused on an even older population (mean age approximately 76 years). Patient body mass index (BMI) data were fully reported in all studies, with baseline BMI values mostly between 22 and 27 kg/m^2^, indicating that the overall weight of the included population was within the normal range, avoiding potential bias in bone metabolism caused by obesity or thinness. None of the studies reported detailed information on patient ethnicity. Regarding the severity of osteoporosis, most studies used a bone mineral density (BMD) T-score ≤ −2.5 SD as the primary diagnostic criterion, but there were differences among studies in specific descriptions of disease course, fracture history, etc. The duration of interventions, i.e., follow-up time, varied considerably, ranging from a minimum of 3 months [Zhao et al. (29)] to a maximum of 12 months [Nilsson et al. (25), Lambert et al. (27)]. The remaining studies typically had intervention durations of 6 months (4 studies) or 12 weeks (90 days, 1 study). This variation is one of the important reasons for the heterogeneity in subsequent meta-analyses.

Regarding interventions, all studies evaluated the effects of probiotics or synbiotic supplements, but the strains, dosages, and combinations used showed high diversity. The probiotic strains encompass multiple species from the genera *Lactobacillus* and *Bifidobacterium*, such as *L. paracasei*, *L. reuteri*, *L. casei*, *L. plantarum*, *L. acidophilus*, *L. rhamnosus*, and *B. animalis*. The strain dosage ranges from 10ˆ8 to 10ˆ10 CFU/day. Studies such as Jansson et al. (17) and Vanichanont et al. (15) employed multi-strain formulations, while studies such as Zhao et al. (29) and Han et al. (16) used single strains or combinations with calcium and vitamin D. Notably, studies such as Desfita et al. (30) and Morato-Martinez et al. (26) employed synbiotic interventions, which involved adding prebiotics (such as inulin) or various bone-health-related micronutrients (such as calcium, vitamin D, K, C, zinc, and magnesium) to the probiotics. In terms of control settings, eight studies used an inert placebo as a control, while two other studies [Desfita et al. (30), Morato-Martinez et al. (26)] used an equivalent basal nutritional supplement without probiotics as a control to assess the incremental benefits of probiotics themselves. All studies measured a wide range of outcome measures, including bone mineral density (lumbar spine, total hip), biochemical markers of bone turnover (osteocalcin, CTX, alkaline phosphatase, osteoprotegerin), parathyroid hormone, and adverse events, providing a rich data foundation for this meta-analysis to comprehensively assess the impact of probiotics/synbiotics on bone metabolism.

### 3.2 Change in BMD of lumbar spine

**FIGURE 3:**
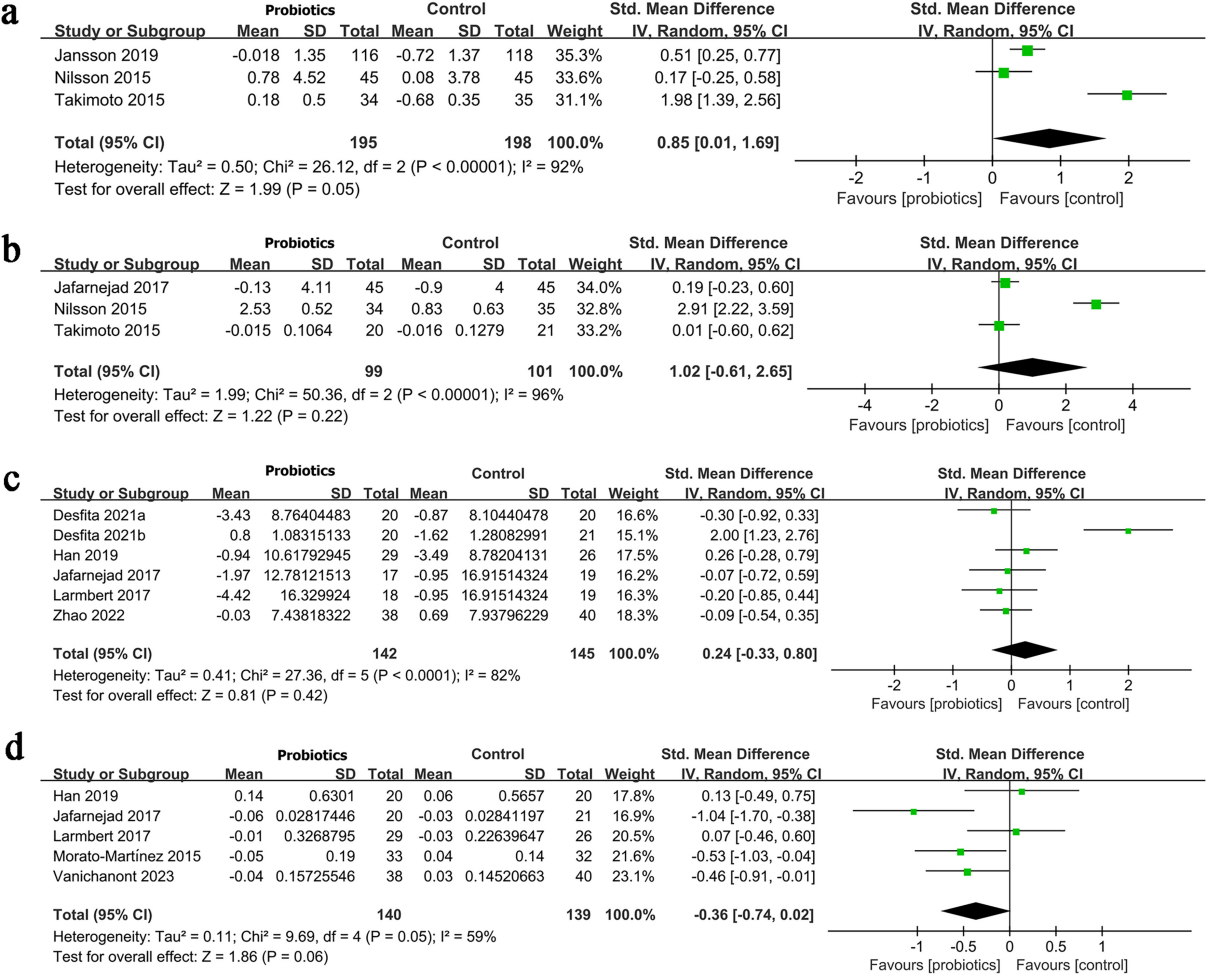
Forest plots of panel **(a)** change in BMD of lumbar spine, **(b)** change in BMD of total hip, **(c)** change in osteocalcin, **(d)** change in CTX.

Results on lumbar spine BMD change were synthesized from 3 RCTs involving 393 patients. Meta-analysis showed a significantly greater increase in lumbar spine BMD in the probiotic/synbiotic group (SMD: 0.85; 95% CI: 0.01, 1.69; *P* = 0.049), with considerable heterogeneity (I^2^ = 92%, *P* < 0.00001) (Figure 3a).

### 3.3 Change in BMD of total hip

The change in total hip BMD was synthesized from three RCTs involving 200 patients. No significant difference between the groups (SMD: 1.02; 95% CI: −0.61, 2.65; *P* = 0.22), with high heterogeneity (I^2^ = 96%, *P* < 0.00001) (Figure 3b).

### 3.4 Change in osteocalcin

The change in osteocalcin was synthesized from six RCTs involving 287 patients. No significant difference found between two groups (SMD: 0.24; 95% CI: −0.33, 0.80; *P* = 0.42), with high heterogeneity (I^2^ = 82%, *P* < 0.0001) (Figure 3c).

### 3.5 Change in CTX

The change in CTX was synthesized from five RCTs involving 279 patients. No significant difference existed between two groups (SMD: −0.36; 95% CI: −0.74, 0.02; *P* = 0.06), with moderate heterogeneity (I^2^ = 59%, *P* = 0.05) (Figure 3d).

### 3.6 Change in parathyroid hormone

**FIGURE 4:**
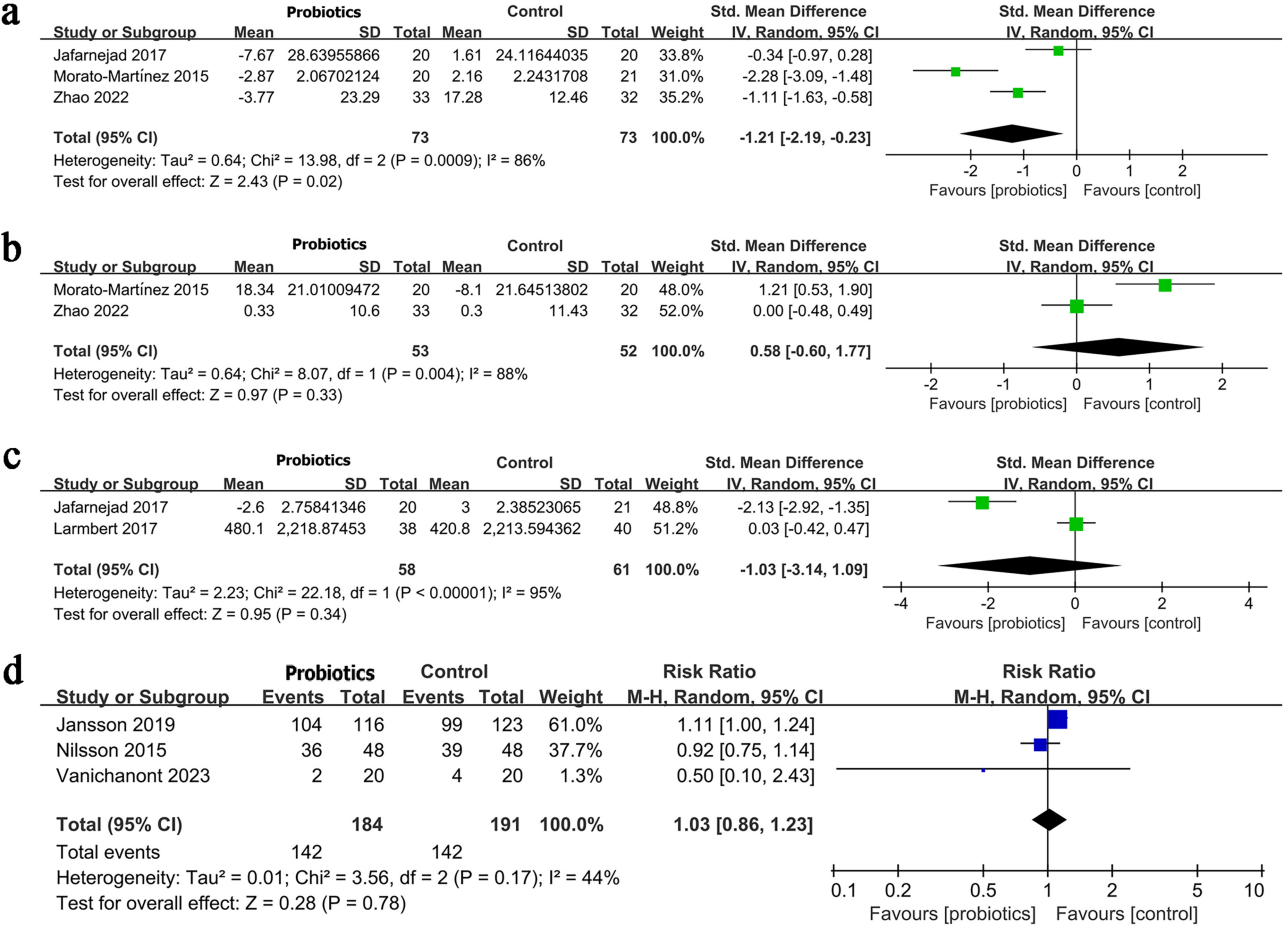
Forest plots of panel **(a)** change in parathyroid hormone, **(b)** change in alkaline phosphatase, **(c)** change in osteoprotegerin, **(d)** any adverse event.

The change in parathyroid hormone was synthesized from three RCTs involving 146 patients. Meta-analysis showed a significantly greater reduction in parathyroid hormone levels in the probiotic/synbiotic group (SMD: −1.21; 95% CI: −2.19, −0.23; *P* = 0.02), with high heterogeneity (I^2^ = 86%, *P* = 0.0009) (Figure 4a).

### 3.7 Change in alkaline phosphatase

The change in alkaline phosphatase was synthesized from two RCTs involving 105 patients. No significant difference between the groups (SMD: 0.58; 95% CI: −0.60, 1.77; *P* = 0.33), with high heterogeneity (I^2^ = 88%, *P* = 0.004) (Figure 4b).

### 3.8 Change in osteoprotegerin

The change in osteoprotegerin was synthesized from two RCTs involving 119 patients. No significant difference (SMD: −1.03; 95% CI: −3.14, 1.09; *P* = 0.34), with very high heterogeneity (I^2^ = 95%, *P* < 0.00001) (Figure 4c).

### 3.9 Any adverse event

The risk of any adverse event was synthesized from three RCTs involving 375 patients. Meta-analysis showed no significant difference between the groups (RR: 1.03; 95% CI: 0.86, 1.23; *P* = 0.78), with moderate heterogeneity (I^2^ = 44%, *P* = 0.17) (Figure 4d).

### 3.10 Sensitivity analysis and publication bias

We conducted sensitivity analysis for the change in lumbar spine BMD, total hip BMD, osteocalcin, CTX, parathyroid hormone, and adverse events to assess the impact of each RCT on the overall SMD or RR by sequentially excluding individual RCTs. The analysis showed that the metrics remained stable after removing each RCT for total hip BMD (Supplementary Figure 1a), osteocalcin (Supplementary Figure 1b), and adverse events (Supplementary Figure 1c). For lumbar spine BMD (Supplementary Figure 1d), Jansson et al. (17) and Nilsson et al. (25) were the main sources of instability. Removing these two studies significantly altered the direction of the combined effect size, indicating that their smaller effect sizes significantly influenced the overall results. For CTX (Supplementary Figure 1e), removing either the Han et al. (16) study or the Lambert et al. (27) study led to significant changes in the effect size and its significance. For parathyroid hormone (Supplementary Figure 1f), removing either the Morato-Martínez et al. (26) study or the Zhao et al. (29) study significantly altered the size and confidence interval of the combined effect size. No significant asymmetry was observed in the funnel plots for lumbar spine BMD (Supplementary Figure 2a), total hip BMD (Supplementary Figure 2b), osteocalcin (Supplementary Figure 2c), CTX (Supplementary Figure 2d), parathyroid hormone (Supplementary Figure 2e), and adverse events (Supplementary Figure 2f).

### 3.11 GRADE rating

Based on the GRADE ratings, this study assessed the quality of evidence for multiple outcome measures. The results showed that the GRADE ratings for changes in lumbar spine bone mineral density (BMD), total hip bone mineral density, osteocalcin, CTX, parathyroid hormone, alkaline phosphatase, and osteoprotegerin were all low. This was primarily due to significant inconsistencies and imprecision in these measures, although the risk of bias and indirectness were not severe, and publication bias was neither detected nor assessed. However, for any adverse event, the GRADE rating was moderate because its inconsistencies were not severe, but other aspects, such as imprecision, still presented limitations. Overall, the quality of evidence was generally low, and the findings should be interpreted with caution. Detailed GRADE classification results are shown in Table 2.

**TABLE 2:** GRADE rating of each outcome.

No. of studies	Outcomes	Risk of bias	Inconsistency	Indirectness	Imprecision	Publication bias	Plausible confounding	Magnitude of effect	Dose-response gradient	GRADE
3	Change in BMD of lumbar spine	No serious risk	Serious inconsistency	No serious indirectness	Serious imprecision	Undetected	Would not reduce effect	No	No	Low
3	Change in BMD of total hip	No serious risk	Serious inconsistency	No serious indirectness	Serious imprecision	Undetected	Would not reduce effect	No	No	Low
6	Change in osteocalcin	No serious risk	Serious inconsistency	No serious indirectness	Serious imprecision	Undetected	Would not reduce effect	No	No	Low
5	Change in CTX	No serious risk	Serious inconsistency	No serious indirectness	Serious imprecision	Undetected	Would not reduce effect	No	No	Low
3	Change in parathyroid hormone	No serious risk	Serious inconsistency	No serious indirectness	Serious imprecision	Undetected	Would not reduce effect	No	No	Low
2	Change in alkaline phosphatase	No serious risk	Serious inconsistency	No serious indirectness	Serious imprecision	NA	Would not reduce effect	No	No	Low
2	Change in osteoprotegerin	No serious risk	Serious inconsistency	No serious indirectness	Serious imprecision	NA	Would not reduce effect	No	No	Low
3	Any adverse event	No serious risk	No serious inconsistency	No serious indirectness	Serious imprecision	Undetected	Would not reduce effect	No	No	Moderate

## 4 Discussion

This study summarized data from 10 recent RCTs, finding that probiotic/synbiotic intervention significantly improves lumbar spine BMD in osteoporosis patients but has no effect on total hip BMD, consistent with Zeng et al.’s meta-analysis (18). However, sensitivity analysis showed that the improvement in lumbar spine BMD was unstable and largely influenced by one study. This indicates that current results do not strongly support probiotics as a significant treatment for osteoporosis. Larger RCTs with bigger sample sizes are needed to confirm these findings. Furthermore, the high heterogeneity observed in this study was primarily due to significant differences among the included studies, including but not limited to the types of probiotic/synbiotic strains used, their combinations, doses, duration of intervention, baseline characteristics of the study population (e.g., age, sex distribution, and osteoporosis severity), and subtle differences in measurement methods. Given the limited number of studies evaluating each specific outcome measure (e.g., only three studies for lumbar spine BMD), meaningful subgroup analyses to fully explore the sources of heterogeneity were not feasible at this stage, which is indeed a significant limitation. Additionally, based on Zeng et al.’s meta-analysis (18), this study found that probiotic/synbiotic intervention significantly reduces parathyroid hormone levels, which may help explain their potential role in protecting against bone loss. However, due to the small sample size, further research is needed to confirm this conclusion.

Probiotics have been reported to cause adverse reactions, such as dry stools and abdominal distension, with long-term and large-scale use potentially leading to constipation (31, 32). In this study, the duration of probiotic treatment varied, with the longest lasting 12 months. While it is unclear whether the adverse reactions reported in the literature are directly caused by probiotics, the drug safety study found no significant difference in overall adverse reactions. None of the studies reported serious adverse reactions related to probiotic treatment. Furthermore, the number of participants who withdrew due to intolerance to probiotic side effects was similar in both groups, indicating good tolerance and safety of oral probiotics. Probiotic metabolites, especially short-chain fatty acids (SCFAs), are considered potential effectors of probiotics on bones, but SCFAs are also associated with the pathogenesis of hepatic encephalopathy (33). Long-term probiotic treatment may increase SCFAs and worsen hepatic coma in patients with liver disease. Some reports suggest that SCFAs could also contribute to experimental hepatic encephalopathy in individuals without liver disease (34). These findings suggest that probiotic safety should be interpreted with caution, especially regarding the use of oral probiotics in patients with low immunity or other diseases and complications, requiring further observation and evaluation.

It has long been recognized that the gastrointestinal system plays a key role in bone homeostasis by regulating calcium absorption. Recent studies, however, have highlighted the primary role of the gut microbiota in regulating bone remodeling (35). Research has clearly shown that the gut influences bone health, particularly through the regulation of mineral absorption, including calcium, phosphorus, and magnesium, which are essential for healthy bones (36). In addition, gut-derived factors, such as incretin and serotonin, also influence bone turnover to varying degrees (37). Research has demonstrated the gut microbiome’s role in regulating bone physiology through studies on the effects of probiotics in germ-free mice (38). In these studies, germ-free mice, conventional mice, and germ-free mice colonized with normal microbiota were used to investigate the role of the gut microbiota in bone. The results showed that germ-free mice had significantly higher bone mass, fewer osteoclasts per bone surface, and a lower number of CD4+ T cells and osteoclast precursors in the bone marrow compared to conventional mice. However, the exact role of the microbiota in bone development remains inconclusive, as some studies have shown no significant difference in bone density between conventional and germ-free mice (39). This suggests that the impact of the microbiota on bone health is complex and requires long-term validation.

Studies have found that *Lactobacillus reuteri* reduces the expression of the inflammatory cytokine TNF-α in the jejunum and ileum, leading to a significant increase in femoral and vertebral trabecular density and trabecular number compared to the untreated control group (40). No difference was observed in serum tartrate-resistant acid phosphatase levels. This study further supports that *Lactobacillus reuteri* benefits bone density. In a study on the mechanism by which intestinal microbiota affects bone, a mouse model showed that yogurt containing *Lactobacillus casei*, *Lactobacillus reuteri*, and *Lactobacillus gasseri* increased calcium absorption in rats, with significantly higher bone mineral content in the experimental group (41). However, further research is needed to identify the specific microbiota that benefit bone health. Modifying the intestinal microbiota through probiotics may, therefore, be a feasible therapeutic strategy to regulate bone remodeling in conditions leading to bone loss and osteoporosis, effectively aiding in the treatment of osteoporosis.

We acknowledge several limitations of this meta-analysis. First, some of the 10 included RCTs did not report detailed blinding procedures, which may affect the reliability of the evidence. Second, the intervention measures in the included RCTs varied (e.g., different probiotic/synbiotic types, doses, and intervention durations), which may explain the significant heterogeneity and instability observed. Third, due to the limited number of studies, this meta-analysis could not gather sufficient data to analyze the effects of probiotic/synbiotic interventions on motor function, quality of life, and other outcomes in osteoporosis patients, which warrants further research. Additionally, due to data limitations, subgroup analysis based on factors such as probiotic types, doses, medication frequency, duration, age, and race were not possible, leaving the potential influence of these factors unclear. Fourth, most of the included studies are from Europe and Asia, with a lack of data from the Americas, Africa, and other regions. Therefore, it is uncertain whether the findings of this study are generalizable worldwide. Finally, although the improvement in lumbar spine BMD reached statistical significance (*P* = 0.049), the lower limit of the 95% confidence interval for the effect estimate was very close to the no effect line, indicating considerable uncertainty in the results. This reflects the instability revealed by sensitivity analyses and the significant heterogeneity. Unfortunately, due to differences in reporting among the original studies, the limited number of studies, and high heterogeneity, we were unable to reliably estimate the mean absolute change in lumbar spine BMD and its potential significance in reducing fracture risk. Therefore, the current results do not strongly support the use of probiotics as a primary treatment for osteoporosis, and larger studies are needed to validate this finding and assess its clinical relevance. Despite these limitations, this meta-analysis updates the analysis of high-quality published RCTs and further validates the efficacy and safety of probiotics and their products in patients with osteoporosis, building on previous studies.

## 5 Conclusion

This study found that probiotics/synbiotics supplementation may be a safe and effective adjunctive treatment for osteoporosis, improving bone density without increasing adverse reactions. However, due to the limitations of this study, including the small number of studies, significant heterogeneity, and potential instability, larger, multicenter RCTs are needed to further confirm the therapeutic effect and safety of probiotics and their products in osteoporosis patients.

## Data Availability

The original contributions presented in this study are included in this article/Supplementary material, further inquiries can be directed to the corresponding authors.

## Supplementary material

Supplementary Figure 1 Sensitivity analysis of panel **(a)** change in BMD of total hip, **(b)** change in osteocalcin, **(c)** and any adverse event, **(d)** change in BMD of lumbar spine, **(e)** change in CTX, and **(f)** change in parathyroid hormone.

Supplementary Figure 2 Funnel plots of panel **(a)** change in BMD of lumbar spine, **(b)** change in BMD of total hip, **(c)** change in osteocalcin, **(d)** change in CTX, **(e)** change in parathyroid hormone, and **(f)** any adverse event.

## References

[1] Luo J Li L Shi W Xu K Shen Y Dai B. Oxidative stress and inflammation: roles in osteoporosis. *Front Immunol.* (2025) 16:1611932. 10.3389/fimmu.2025.1611932 40873591 PMC12379731

[2] Castellani C De Martino E Scapato P. Osteoporosis: focus on bone remodeling and disease types. *BioChem.* (2025) 5:31. 10.3390/biochem5030031

[3] Kenzhegazova G Baspakova A Umbetova A. Osteoporosis: a comprehensive review of epidemiology, impact on quality of life, and treatment strategies. *West Kazakhstan Med J.* (2025) 67:177–87. 10.18502/wkmj.v67i2.16570

[4] Bulbuli N. *The Prevalence, Risk Factors, Diagnosis & Treatment of Osteoporosis in Bangladesh.* Dhaka: BRAC University (2025).

[5] Wang D Shen J Wang Y Cui H Li Y Zhou L Mechanisms of Ferroptosis in bone disease: a new target for osteoporosis treatment. *Cell Signal.* (2025) 127:111598. 10.1016/j.cellsig.2025.111598 39788305

[6] Lamy O Everts-Graber J Rodriguez E. Denosumab for osteoporosis treatment: when, how, for whom, and for how long. A pragmatical approach. *Aging Clin Exp Res.* (2025) 37:1–11. 10.1007/s40520-025-02991-z 40055268 PMC11889064

[7] Mandal M Amjad H Oyovwi J Olaniyi A Fayyaz M. Atypical femoral fracture secondary to long-term bisphosphonate use: a case report. *Cureus.* (2025) 17:e90611. 10.7759/cureus.90611 40978969 PMC12449035

[8] Jia J Tian C Han W Ma Q. Real-world assessment of thromboembolic risk associated with tamoxifen. *Sci Rep.* (2025) 15:27820. 10.1038/s41598-025-13585-0 40739320 PMC12310949

[9] Nayak S Greenspan SL. A systematic review and meta-analysis of sequential treatment strategies for osteoporosis. *Osteoporos Int.* (2025): 10.1007/s00198-025-07717-5 Online ahead of print 41105226

[10] Lerner E MacLean K Colón-Emeric C Berry S. Enabling osteoporosis treatment in post-acute care: an algorithm for providers. *Geriatr Nurs.* (2025) 61:228–30. 10.1016/j.gerinurse.2024.10.068 39566233 PMC11805628

[11] Qu X Xie Z Zhang J Huang Y Zhao R Li N Regulating mitochondrial aging via targeting the gut-bone axis in BMSCs with oral hydrogel microspheres to inhibit bone loss. *Small.* (2025) 21:e2409936. 10.1002/smll.202409936 39629509

[12] Mazziotta C Tognon M Martini F Torreggiani E Rotondo J. Probiotics mechanism of action on immune *cells* and beneficial effects on human health. *Cells.* (2023) 12:184. 10.3390/cells12010184 36611977 PMC9818925

[13] Ranjan A. *The Use of Probiotics, Prebiotics, and Synbiotics as an Alternative to Antibiotics. Alternatives to Antibiotics: Recent Trends and Future Prospects.* Berlin: Springer (2022). p. 449–65.

[14] Hossain M Sultana T Moon J Moon G Jeong J. Anti-osteoporotic potential of a probiotic mixture containing Limosilactobacillus reuteri and Weissella cibaria in ovariectomized rats. *Sci Rep.* (2025) 15:18586. 10.1038/s41598-025-02089-6 40425630 PMC12116856

[15] Vanitchanont M Vallibhakara S Sophonsritsuk A Vallibhakara O. Effects of multispecies probiotic supplementation on serum bone turnover markers in postmenopausal women with osteopenia: a randomized, double-blind, placebo-controlled trial. *Nutrients.* (2024) 16:461. 10.3390/nu16030461 38337745 PMC10857023

[16] Han H Kim J Choi Y Lee K Kwon T Kim S. Effect of Lactobacillus fermentum as a probiotic agent on bone health in postmenopausal women. *J Bone Metab.* (2022) 29:225–33. 10.11005/jbm.2022.29.4.225 36529865 PMC9760773

[17] Jansson P Curiac D Lazou Ahrén I Hansson F Martinsson Niskanen T Sjögren K Probiotic treatment using a mix of three Lactobacillus strains for lumbar spine bone loss in postmenopausal women: a randomised, double-blind, placebo-controlled, multicentre trial. *Lancet Rheumatol.* (2019) 1:e154–62. 10.1016/S2665-9913(19)30068-2 38229392

[18] Zeng L Yu G Yang K Hao W Chen H. The improving effect and safety of probiotic supplements on patients with osteoporosis and osteopenia: a systematic review and meta-analysis of 10 randomized controlled trials. *Evid Based Complement Alternat Med.* (2021) 2021:9924410. 10.1155/2021/9924410 34349831 PMC8328694

[19] Page M McKenzie J Bossuyt P Boutron I Hoffmann T Mulrow C The PRISMA 2020 statement: an updated guideline for reporting systematic reviews. *BMJ.* (2021) 372:n71. 10.1136/bmj.n71 33782057 PMC8005924

[20] Cumpston M Li T Page M Chandler J Welch V Higgins J Updated guidance for trusted systematic reviews: a new edition of the cochrane handbook for systematic reviews of interventions. *Cochrane Database Syst Rev.* (2019) 10:ED000142. 10.1002/14651858.ED000142 31643080 PMC10284251

[21] Higgins J Thompson S. Quantifying heterogeneity in a meta-analysis. *Stat Med.* (2002) 21:1539–58. 10.1002/sim.1186 12111919

[22] Egger M Davey Smith G Schneider M Minder C. Bias in meta-analysis detected by a simple, graphical test. *BMJ.* (1997) 315:629–34. 10.1136/bmj.315.7109.629 9310563 PMC2127453

[23] Guyatt G Oxman A Akl E Kunz R Vist G Brozek J GRADE guidelines: 1. Introduction-GRADE evidence profiles and summary of findings tables. *J Clin Epidemiol.* (2011) 64:383–94. 10.1016/j.jclinepi.2010.04.026 21195583

[24] Takimoto T Hatanaka M Hoshino T Takara T Tanaka K Shimizu A Effect of Bacillus subtilis C-3102 on bone mineral density in healthy postmenopausal Japanese women: a randomized, placebo-controlled, double-blind clinical trial. *Biosci Microbiota Food Health.* (2018) 37:87–96. 10.12938/bmfh.18-006 30370192 PMC6200670

[25] Nilsson A Sundh D Bäckhed F Lorentzon M. Lactobacillus reuteri reduces bone loss in older women with low bone mineral density: a randomized, placebo-controlled, double-blind, clinical trial. *J Intern Med.* (2018) 284:307–17. 10.1111/joim.12805 29926979

[26] Morato-Martínez M López-Plaza B Santurino C Palma-Milla S Gómez-Candela CA. Dairy product to reconstitute enriched with bioactive *Nutrients* stops bone loss in high-risk menopausal women without pharmacological treatment. *Nutrients.* (2020) 12:2203. 10.3390/nu12082203 32722015 PMC7468696

[27] Lambert M Thybo C Lykkeboe S Rasmussen L Frette X Christensen L Combined bioavailable isoflavones and probiotics improve bone status and estrogen metabolism in postmenopausal osteopenic women: a randomized controlled trial. *Am J Clin Nutr.* (2017) 106:909–20. 10.3945/ajcn.117.153353 28768651

[28] Jafarnejad S Djafarian K Fazeli M Yekaninejad M Rostamian A Keshavarz S. Effects of a multispecies probiotic supplement on bone health in osteopenic postmenopausal women: a randomized, double-blind, controlled trial. *J Am Coll Nutr.* (2017) 36:497–506. 10.1080/07315724.2017.1318724 28628374

[29] Zhao F Guo Z Kwok L Zhao Z Wang K Li Y Bifidobacterium lactis Probio-M8 improves bone metabolism in patients with postmenopausal osteoporosis, possibly by modulating the gut microbiota. *Eur J Nutr.* (2023) 62:965–76. 10.1007/s00394-022-03042-3 36334119

[30] Desfita S Sari W Yusmarini Y Pato U Zakłos-Szyda M Budryn G. Effect of fermented soymilk-honey from different probiotics on osteocalcin level in menopausal women. *Nutrients.* (2021) 13:3581. 10.3390/nu13103581 34684581 PMC8541044

[31] Gill B Wessels J Hayes C Ratcliffe J Wokuri J Ball E Feasibility, safety and tolerability of estrogen and/or probiotics for improving vaginal health in Canadian African, Caribbean, and black women: a pilot phase 1 clinical trial. *PLoS One.* (2025) 20:e0315576. 10.1371/journal.pone.0315576 39836666 PMC11750099

[32] García G Soto J Díaz A Barreto J Soto C Pérez A Clinical and in vitro safety of Heyndrickxia coagulans AO 1167B: a double-blind, placebo-controlled trial. *Microorganisms.* (2024) 12:2584. 10.3390/microorganisms12122584 39770785 PMC11677179

[33] Liu T Chen G Lin C Tsai T Cheng M. Lactobacillus plantarum TWK10 relieves loperamide-induced constipation in rats fed a high-fat diet via modulating enteric neurotransmitters, short-chain fatty acids and gut microbiota. *Food Funct.* (2025) 16:181–94. 10.1039/d4fo02270j 39641806

[34] Baraldi M Zeneroli M Ventura E Vezzelli C. An increase in cerebral benzodiazepine receptors induced by a subacute administration of ammonia, mercaptans and short-chain fatty acids in rats. *Clin Sci.* (1987) 73:669–71. 10.1042/cs0730669 2826071

[35] He Y Chen Y. The potential mechanism of the microbiota-gut-bone axis in osteoporosis: a review. *Osteoporos Int.* (2022) 33:2495–506. 10.1007/s00198-022-06557-x 36169678

[36] Wang J Xing F Sheng N Xiang Z. Associations of the dietary magnesium intake and magnesium depletion score with osteoporosis among American adults: data from the national health and nutrition examination survey. *Front Nutr.* (2022) 9:883264. 10.3389/fnut.2022.883264 35711538 PMC9194572

[37] Fujimoto K Yamanouchi K Kishi T. [Role of the gastrointestinal tract on therapeutic approach to obesity]. *Nihon Shokakibyo Gakkai Zasshi.* (2021) 118:500–4. 34108348 10.11405/nisshoshi.118.500

[38] Sjögren K Engdahl C Henning P Lerner U Tremaroli V Lagerquist M The gut microbiota regulates bone mass in mice. *J Bone Miner Res.* (2012) 27:1357–67. 10.1002/jbmr.1588 22407806 PMC3415623

[39] Aboushaala K Wong A Barajas J Lim P Al-Harthi L Chee A The human microbiome and its role in musculoskeletal disorders. *Genes.* (2023) 14:1937. 10.3390/genes14101937 37895286 PMC10606932

[40] Behera J Ison J Voor M Tyagi N. Probiotics stimulate bone formation in obese mice via histone methylations. *Theranostics.* (2021) 11:8605–23. 10.7150/thno.63749 34373761 PMC8344023

[41] Seijo M Bonanno M Vénica C Marotte C Pita Martín de Portela ML Bergamini CV A yoghurt containing galactooligosaccharides and having low-lactose level improves calcium absorption and retention during growth: experimental study. *Int J Food Sci Technol.* (2022) 57:48–56. 10.1111/ijfs.15212

