# Supplementary figures and images for: Efficacy and safety of probiotic/synbiotic supplementation for osteoporosis: a meta-analysis of randomized controlled trials
Source: Front Med (Lausanne). 2026 Feb 3;13:1731528. doi: 10.3389/fmed.2026.1731528 (PMC12909203; doi:10.3389/fmed.2026.1731528)

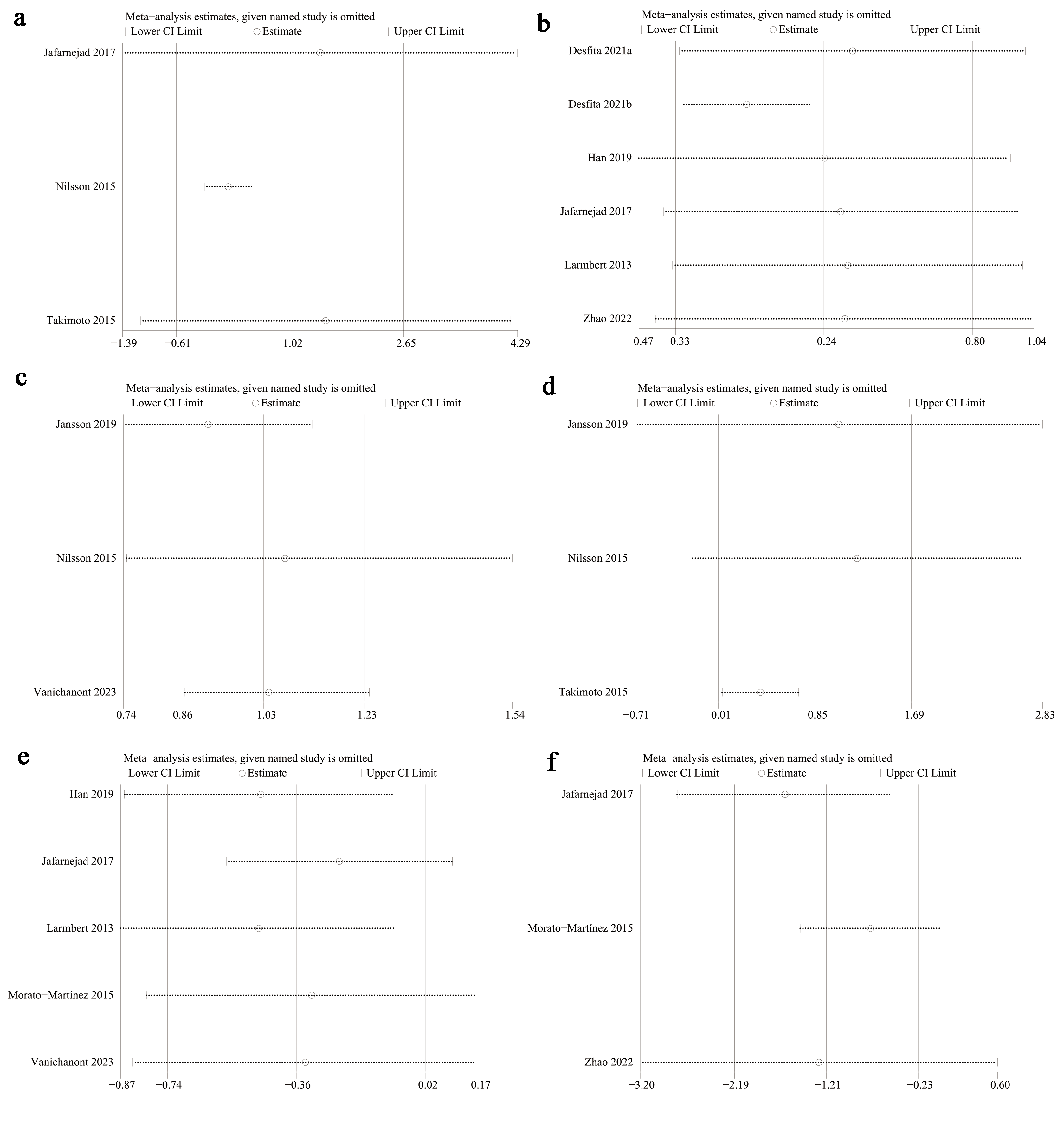

Supplement: Supplementary Figure 1 — Sensitivity analysis of panel (a) change in BMD of total hip, (b) change in osteocalcin, (c) and any adverse event, (d) change in BMD of lumbar spine, (e) change in CTX, and (f) change in parathyroid hormone. [file Image_1.tif]

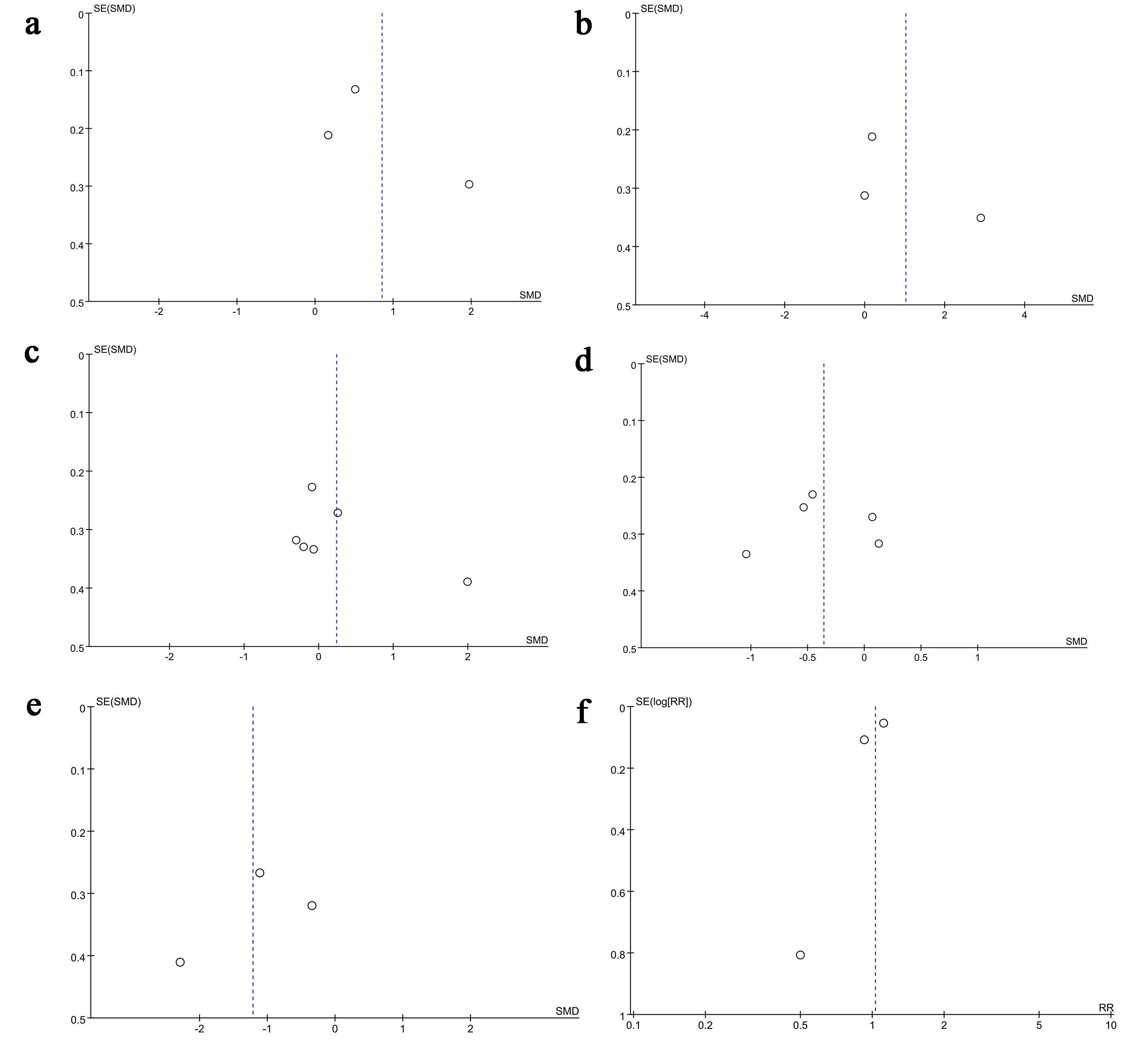

Supplement: Supplementary Figure 2 — Funnel plots of panel (a) change in BMD of lumbar spine, (b) change in BMD of total hip, (c) change in osteocalcin, (d) change in CTX, (e) change in parathyroid hormone, and (f) any adverse event. [file Image_2.tif]
