# Supplementary material for: Efficacy and safety of probiotic/synbiotic supplementation for osteoporosis: a meta-analysis of randomized controlled trials
Source: Front Med (Lausanne). 2026 Feb 3;13:1731528. doi: 10.3389/fmed.2026.1731528 (PMC12909203; doi:10.3389/fmed.2026.1731528)
Supplement: Supplementary file 3 [file Data_Sheet_1.doc]

**Table S1 Literature Search Strategy**

PubMed-243

**((("Probiotics"[Mesh]) OR (Probiotic)) OR (("Synbiotics"[Mesh]) OR (Synbiotic))) AND (("Osteoporosis"[Mesh]) OR ((((((((((Osteoporoses) OR (Post-Traumatic Osteoporoses)) OR (Post-Traumatic Osteoporosis)) OR (Senile Osteoporoses)) OR (Senile Osteoporosis)) OR (Age-Related Bone Loss)) OR (Age-Related Bone Losses)) OR (Age-Related Osteoporosis)) OR (Age Related Osteoporosis)) OR (Age-Related Osteoporoses)))**

**Embase-577**


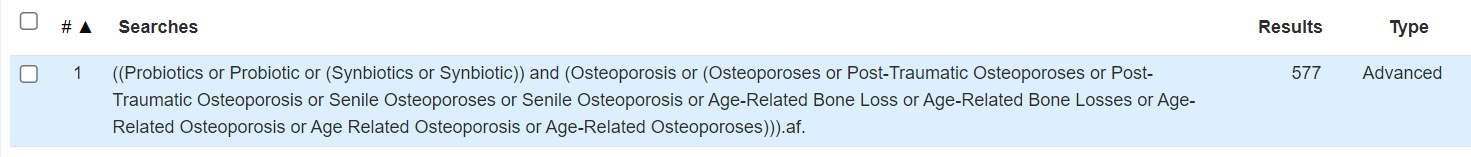


Cochrone-41


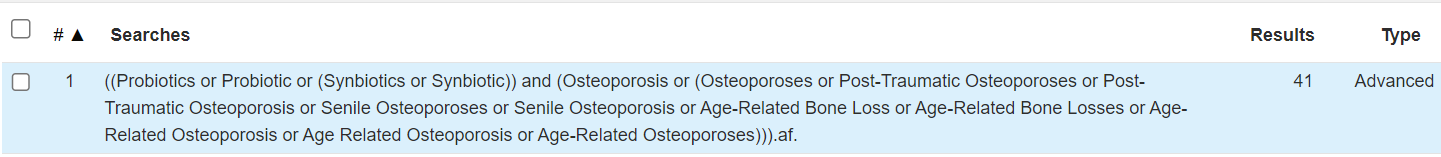


**Web of science-224**

**(((Probiotics) OR (Probiotic)) OR ((Synbiotics) OR (Synbiotic))) AND ((Osteoporosis) OR ((((((((((Osteoporoses) OR (Post-Traumatic Osteoporoses)) OR (Post-Traumatic Osteoporosis)) OR (Senile Osteoporoses)) OR (Senile Osteoporosis)) OR (Age-Related Bone Loss)) OR (Age-Related Bone Losses)) OR (Age-Related Osteoporosis)) OR (Age Related Osteoporosis)) OR (Age-Related Osteoporoses)))** (Topic)
